# Diabetes, cardiac disorders and asthma as risk factors for severe organ involvement among adult dengue patients: A matched case-control study

**DOI:** 10.1038/srep39872

**Published:** 2017-01-03

**Authors:** Junxiong Pang, Jung Pu Hsu, Tsin Wen Yeo, Yee Sin Leo, David C. Lye

**Affiliations:** 1 Institute of Infectious Diseases and Epidemiology, Tan Tock Seng Hospital, Singapore; 2 Saw Swee Hock School of Public Health, National University of Singapore, Singapore; 3 Lee Kong Chian School of Medicine, Nanyang Technological University, Singapore; 4 Yong Loo Lin School of Medicine, National University of Singapore, Singapore

## Abstract

Progression to severe organ involvement due to dengue infection has been associated with severe dengue disease, intensive care treatment, and mortality. However, there is a lack of understanding of the impact of pre-existing comorbidities and other risk factors of severe organ involvement among dengue adults. The aim of this retrospective case-control study is to characterize and identify risk factors that predispose dengue adults at risk of progression with severe organ involvement. This study involved 174 dengue patients who had progressed with severe organ involvement and 865 dengue patients without severe organ involvement, matched by the year of presentation of the cases, who were admitted to Tan Tock Seng Hospital between year 2005 and 2008. Age group of 60 years or older, diabetes, cardiac disorders, asthma, and having two or more pre-existing comorbidities were independent risk factors of severe organ involvement. Abdominal pain, clinical fluid accumulation, and hematocrit rise and rapid platelet count drop at presentation were significantly associated with severe organ involvement. These risk factors, when validated in a larger study, will be useful for triage by clinicians for prompt monitoring and clinical management at first presentation, to minimize the risk of severe organ involvement and hence, disease severity.

Dengue is currently the most important mosquito-borne viral pathogen affecting humans, and is emerging as a major threat to global health. It results in a significant public health and economic burden in the endemic regions^1^,^2^, particularly in the World Health Organization (WHO) South-East Asia and Western Pacific Regions, accounting for nearly 75% of the current global dengue disease burden^3^. Best estimates indicate that some 3 billion people live in these tropical and subtropical regions where they are at risk of infection and that around 96 million symptomatic episodes and approximately 20,000 deaths occur each year^4^. The high burden was largely due to a lack of licensed vaccines then and specific therapies although there have been enormous research efforts focusing on these two areas. The ongoing implementation of a recently licensed Denvaxia^®^ tetravalent dengue vaccine across various dengue-endemic countries^5^ may help to reduce these dengue burden in the near future.

Dengue is caused by infection with any one of four related dengue virus (DENV) serotypes that belongs to the genus Flavivirus. Although most dengue infections are asymptomatic, patients can present with a wide spectrum of clinical symptoms ranging from mild febrile illness through to severe manifestations of bleeding, organ involvement, and hypovolemic shock due to a systemic vascular leak syndrome. However, with good supportive care (primarily judicious use of parenteral fluid therapy to offset plasma volume losses due to leakage), mortality rates of less than 1% are possible even among severe cases^6^,^7^.

Although the vast majority of symptomatic infections do not progress to severe disease, areas of high dengue transmission can have seasonal epidemics, which can quickly overwhelm health service capacity, particularly in the tertiary care settings. Thus, the ability to identify patients at high risk of severe disease progression, who are likely to benefit from close observation and early intervention with supportive therapy, has become the focus of intense research efforts in recent years. In recognizing the changing needs and increasing trends of difficulty in clinical application on the ground, the World Health Organization revised the previous dengue classifications^8^ (Dengue fever [DF], Dengue hemorrhagic fever [DHF] and dengue shock syndrome [DSS]) into two major entities, DF and severe dengue (SD), where DF comprises two subgroups of those with or without warning signs^9^ to help clinicians identify patients who are likely to develop complications during the critical phase of the illness^10^. However, the utility of warnings signs were limited among adult patients^11^,^12^, probably due to the small number of adult patients progressing to severe disease in the study that derived these warning signs^13^.

One of the major dengue complications of concern is the progression to severe organ involvement, which has been associated with SD, intensive care treatment, and fatality^13^,^14^,^15^,^16^,^17^. Dengue associated severe organ involvement were cardiovascular^18^, hepatic^14^,^19^,^20^, renal^21^,^22^, as well as central nervous systems^23^,^24^, and to some extent, respiratory^25^,^26^,^27^ and muscular systems^28^,^29^. Different dengue serotypes may have different severe organ involvements^30^. Moreover, severe organ involvement has been associated with adult dengue, and are likely to occur late in the disease course, but occurs rapidly when it happens, with limited time for optimal clinical management^3^,^10^. Increasingly, pre-existing comorbidities such as diabetes have been shown to be a significant public health burden in the WHO Western Pacific and South-East Asia regions^31^, which are also dengue endemic regions. However, there is still limited understanding of the impact of pre-existing comorbidities^32^ and other early risk factors among dengue adults with severe organ involvement. Therefore, the aim of this retrospective case-control study is to characterize and identify risk factors that are useful to stratify adult dengue patients at a tertiary hospital at risk of progression with severe organ involvement.

## Results

From year 2005 to 2008, there were about 6,300 dengue patients admitted into Tan Tock Seng Hospital. Among which, there were 174 (2.76%) dengue patients who had progressed with severe organ involvement. Among the cases, there were 106 (61%) who had hepatic involvement, 60 (34.5%) had renal involvement, 23 (13.2%) had neurological involvement, and 15 (8.6%) had both renal and liver involvement. The median age of these cases was 37.5 years old (Inter-quartile range: 27–52). Among these cases, 84 (48.3%) were female, 114 (65.5%) of Chinese ethnicity, and median duration of fever at presentation was 5 days (IQR: 4–6 days). In addition, 55 (31.6%) of these cases presented with pre-existing illnesses (Table 1).

### Age and existing co-morbidities as risk factors of severe organ involvement

Dengue patients within age group of 60 years of age and older had 2.75 times higher (AOR: 2.75; 95% CI: 1.3–5.8) risk than age group between 12–29 years of age. Patients who presented with any pre-existing co-morbidity had 1.63 times higher (AOR: 1.63; 95% CI: 1.07–2.49) risk than dengue patients who did not presented with any existing co-morbidity. In addition, dengue patients with two or more existing co-morbidities had 2.90 times higher (95% CI: 1.66–5.07) risk than dengue patients with no existing co-morbidities. Dengue patients with pre-existing diabetes had 2.21 times higher (AOR: 2.21; 95% CI: 1.10–5.02) risk than dengue patients without diabetes. Dengue patients with pre-existing cardiac disorder had 4.3 times higher (AOR: 4.30; 95% CI: 1.45–12.78) risk than dengue patients without cardiac disorders. Dengue patients with pre-existing asthma had 2.14 times higher (AOR: 2.14; 95% CI: 1.04–4.42) risk than dengue patients without asthma (Table 1).

Using these significantly associated co-morbidities as risk factors, the impact of dual pre-existing comorbidities was assessed. Dengue patients with both pre-existing diabetes and cardiac disorders had eight times higher risk of severe organ involvement (AOR: 8.02; 95% CI: 1.40–45.92) than dengue patients with none of these (Table 2). In addition, we observed that dengue patients with both diabetes and hypertension (AOR: 2.68; 95% CI: 1.07–6.68) or both diabetes and hyperlipidemia (AOR: 4.25; 95% CI: 1.34–13.52) or both cardiac disorders and hyperlipidemia (AOR: 5.79; 95% CI: 1.03–32.64) had significant increase in risk compared with dengue patients with only one of the comorbidities or none at all. There was a lack of controls for dual comorbidities involving asthma, and hence, it was excluded (Table 2).

### Clinical severity & management characteristics as risk factors of severe organ involvement

Based on WHO 1997 dengue classifications, dengue patients with initial diagnosis of DHF/DSS had 3.11 times higher (AOR: 3.11; 95% CI: 2–4.85) risk of subsequent severe organ involvement. However, any dengue warning signs category at presentation based on the WHO 2009 dengue classifications was not a risk factor for subsequent severe organ involvement. At discharge, both DHF/DSS and any dengue warning signs category were significantly associated with severe organ involvement. The median length of hospitalization was five days (IQR: 4–7 days) longer than dengue patients without severe organ involvement. Overall, 6.9% of those with severe organ involvement required intensive care admission, compared to none of the dengue patients without severe organ involvement. There were also significantly more dengue patients with severe organ involvement who required intravenous fluid (96%) with larger fluid volume (median: 6 liters; IQR: 3.9–8.7 liters), blood transfusion (5.8%) and platelet transfusion (33.3%) than dengue patients without severe organ involvement. There was no death in both groups (Table 3).

### Signs and symptoms at presentation as risk factors of severe organ involvement

At presentation, abdominal pain (AOR: 2.02; 95% CI: 1.40–2.93), clinical fluid accumulation (AOR: 26.2; 95% CI: 2.51–274.3) and hematocrit rise and rapid platelet count drop (AOR: 6.67; 95% CI: 3.98–11.17) were specific warning signs significantly associated with severe organ progression. Dengue patients with severe organ involvement were significantly associated with nausea or vomiting (AOR: 1.65; 95% CI: 1.08–1.87), but not persistent vomiting at presentation. In addition, plasma leakage at presentation (AOR: 6.19; 95% CI: 4.09–9.40) was observed to be significantly associated with dengue patients with severe organ involvement, but not hemorrhagic manifestations at presentation (Table 4).

### Clinical laboratory characteristics at presentation as risk factors of severe organ involvement

White blood cell (median: 3.3; IQR: 2.1–4.7) and neutrophil counts (median: 61.7; IQR: 50–70) were significantly higher at presentation among dengue patients who progressed with severe organ involvement compared with dengue patients without severe organ involvement. However, lymphocyte (median: 20.2; IQR: 15–28.9) and eosinophil counts (median: 0.1; IQR: 0–0.5) were significantly lower at presentation among dengue patients who progressed with severe organ involvement compared with dengue patients without severe organ involvement. Alanine (median: 771; IQR: 174–1242) and aspartate aminotransferases (median: 91.5; IQR: 91.5–717) were observed to be significantly higher at presentation among dengue patients who progressed with severe organ involvement compared with dengue patients without severe organ involvement (Table 5).

## Discussion

Severe organ involvement has been associated with severe dengue, intensive care requirement, and fatality^13^,^14^,^15^,^16^,^17^. Chronic medical disorders have also been implicated in the development of severe dengue diseases^32^. However, there is still a lack of understanding on the impact of comorbidities on adult dengue patients who progressed with severe organ involvement, which is one of the criteria for severe dengue classification. Identification of these risk factors at presentation may guide early triage and clinical management of adult dengue patients who are at high risk of severe organ involvement.

Age group of 60 years old and above was observed to be an independent risk factor of severe organ involvement. Even after adjusting for the potential confounding effect of pre-existing comorbidities, age group of 60 years old and above has about three times higher risk for severe organ involvement than those aged 12–29 years old. This may not be surprising as elderly were also reported to be an independent risk factor for severe dengue in Thailand^33^, Malaysia^34^, Taiwan^35^ and Singapore^36^, and organ impairment was significantly associated with increased age in Vietnam^37^. This may be due to immunosenescence among elderly, which predisposes them to infectious diseases^38^. Immunosenescence is characterized by reduced natural killer cell cytotoxicity; reduced number and function of dendritic cells in blood; decreased pools of naive T and B cells; and increases in the number of memory and effector T and B cells^39^. In addition, elderly tend to have impaired functional reserve for various organ systems, and with the reported tropism of dengue virus for liver, spleen, lymph node, kidney, bone marrow, lung, thymus and brain^40^, may increase the risk of severe organ impairment.

Co-morbidities such as diabetes mellitus, cardiac disorders and asthma are among the few leading causes of mortality and morbidity in Asia^31^. However, there are still limited reported systematic studies so far to assess the association between these comorbidities and severe organ involvement due to dengue as a form of disease severity^32^. In this study, having any pre-existing comorbidity was observed to be an independent risk factor of severe organ involvement. Similarly, this was reported to be associated with dengue hemorrhagic fever (DHF) and severe dengue^32^. Furthermore, the risk of severe organ involvement was found to be three times higher among patients with at least two pre-existing comorbidities compared to patients with none. This observation suggests that there could be multiplicative effect modification. Diabetes, cardiac disorders and asthma were observed to be independent risk factors of severe organ involvement. Diabetes was well-reported to be a risk factor for dengue severity^41^,^42^,^43^, as well as acute kidney injury^22^. However, the pathophysiology behind diabetes leading to severe organ involvement outcome is not well understood yet, even though numerous studies had suggested that diabetes mellitus can result in immune and endothelial dysfunction^44^,^45^. Pre-existing cardiac disorders have also been reported as a risk factor for dengue fatality^46^. Cardiac output, is one of the major determinants of renal blood flow autoregulation. Renal blood flow is highly regulated to ensure oxygen delivery for normal renal function^47^. If cardiac output is compromised, ischemic and toxic injury to the kidney can occur, resulting in severe impairment^48^ especially when dengue virus has also been reported to affect kidney function^22^. Cardiovascular manifestations due to dengue has been reviewed^18^, however, there was no dengue-related myocarditis reported as disease outcome in this study. Asthma has been reported as a risk factor for DHF^49^,^50^,^51^. It was observed that dengue virus enhancement within peripheral blood leukocytes was more significant among the asthmatic group compared to non-asthmatic group^52^, which may promote higher viral load leading to severe organ involvement. Asthma is a disorder of variable airflow obstruction in association with airway hyper-responsiveness (AHR). T Helper 2 (T_H_2) cell-type cytokines orchestrate the allergic inflammatory cascade that occurs in asthma^53^. Hence, with dengue virus infection, it may potentially activate the allergic inflammatory cascade, that may also result in selective organ damage. On the other hand, as an intrinsic abnormality, impaired TLR3-mediated interferon-β (IFN-β)^54^ and IFN-λ^55^ production by asthmatic epithelial cells may also predispose to dengue virus indirectly^56^,^57^. This allergic inflammatory cascade may also play a role in the development of acute respiratory failure, which was reported to be a risk factor of dengue fatal cases^27^. Future studies will be required to validate this hypothesis.

Interestingly, having two or more comorbidities was observed to be an independent risk factor of severe organ involvement after adjusting for age as potential confounder. So far only diabetes with hypertension was reported as a risk factor of DHF^42^. Similarly, diabetes with hypertension remained an independent risk factor of severe organ involvement. Hypertension was reported to be a risk factor of rhabdomyolysis^28^. In addition, diabetes with hyperlipidemia, diabetes with cardiac disorders and cardiac disorders with hyperlipidemia were observed to be independent risk factors of severe organ involvement. The underlying mechanism of diabetes, hypertension, hyperlipidemia, and cardiac disorders is inter-related and involves endothelial dysfunction^58^ and increased nitric oxide^59^, which has been shown to result in increased ‘cytokine tsunami’^60^ and vascular permeability, leading to DHF^61^. This further suggests that these comorbidities have a major but indirect impact on the dengue disease progression with severe organ involvement^48^, particularly on hepatic, renal and central nervous systems as observed in this study. Future studies focusing on the impact of medications for management of these pre-existing comorbidities and the underlying immunopathogenesis leading to severe organ involvement are needed to provide a better understanding on the impact of the interaction between dengue virus and non-communicable diseases.

Having any dengue warning signs at presentation was not a risk factor as a potential triage for severe organ involvement. However, it was observed in other published studies that any warning signs had very high sensitivity but only modest specificity for predicting DHF and severe dengue progression^11^,^12^. Abdominal pain, clinical fluid accumulation, and hematocrit rise and rapid platelet count drop at presentation were significantly associated with severe organ involvement progression, which were also shown to be associated with severe dengue progression^11^,^13^. Hematocrit rise and rapid platelet count drop and neutrophil count at presentation were associated with intensive care unit admission^62^. Dengue patients with severe organ involvement were significantly associated with nausea or vomiting, but not persistent vomiting at presentation. This may not be a useful risk factor as most dengue patients with mild disease may present with nausea or vomiting^63^. In addition, plasma leakage was observed to be significantly associated with dengue patients with severe organ involvement, but not hemorrhagic manifestations at presentation. This is consistent with the reported observation that plasma leakage has a role in the development of severe organ involvement^64^. However, the underlying mechanism of the role of plasma leakage is not clear and requires further studies.

As this is a retrospective study, the quality of the study was dependent on the quality of the data available and collected. Information bias was minimized by the use of the standardized dengue care path for consistent clinical documentation. Reporting bias was minimized by the fact that patients with comorbidities tend to know their existing conditions and are on regular medications. However, undetected existing comorbidities among the controls could not be excluded in this retrospective study. In addition, there may be selection bias because the subjects were all hospitalized and hence were likely to be more severe and/ or have a more active health seeking behavior who may not represent the general dengue population in the community. We did not have patient-specific dengue serotype data and could only minimize the confounding effect of different serotypes by matching each case to five controls by year of presentation. Lastly, we understand the importance of accounting for prior infection as it is a main risk factor for severe dengue disease. The result of IgG test carried out within seven days of fever onset can be used to classify patients with or without prior infection. From our limited data of potential secondary infections, we showed that secondary infection was not significantly associated with severe organ involvement in adult patients (Table 1). Further studies involving larger number of patients with acute secondary infections are required to confirm this hypothesis.

## Conclusion

Severe organ involvement results in severe dengue disease. Age group of 60 years or older, diabetes, cardiac disorders, asthma, and two or more pre-existing comorbidities were independent risk factors of severe organ involvement. Abdominal pain, clinical fluid accumulation, and hematocrit rise and rapid platelet count drop at presentation were significantly associated with severe organ involvement. With larger prospective studies to validate these findings, they may be useful to guide triage at presentation of adult dengue patients who are at higher risk of severe organ involvement.

## Methods

A retrospective case-control study was conducted using data collected from all adult dengue patients admitted from 1 January 2005 to 31 December 2008 to the Department of Infectious Diseases at Tan Tock Seng Hospital (TTSH). It is the largest hospital in Singapore for the treatment of dengue patients where dengue patients were managed using a standardized dengue care path. Hospital electronic medical records were used for extraction of administrative, laboratory, microbiological and radiological data. Data extraction was performed by medically trained research assistants. Rule-based data validation was performed for the entire data set. In addition, 10% of the cases were randomly selected for repeat data entry by another research assistant; data discrepancy was resolved by independent medical case note review by one of the authors. The extracted data was de-identified in analysis.

Probable dengue patients had positive acute dengue serology, as measured by Dengue Duo IgM & IgG Rapid Strip Test (Panbio Diagnostic, Queensland, Australia), and fulfilled clinical diagnostic criteria of dengue fever by WHO 1997. Confirmed dengue patients had positive dengue polymerase chain reaction (PCR) assay. Patients were classified into the WHO 1997 and 2009 dengue severity categories with available clinical, laboratory and radiological data from the entire clinical course till hospital discharge for inpatients and end of acute follow up for outpatients with strict application of the two WHO classifications. As per previous published study^46^, cardiac disorder is defined ischemic or other heart diseases excluding heart failure and peripheral vascular diseases. Renal disorder is defined as having renal failure, calculi or other kidney diseases

### WHO 1997 Classification

Based on WHO 1997 classification^8^, classification as dengue fever (DF) requires the presence of fever and two or more of the following: headache, retro-orbital pain, myalgia, arthralgia, rash, leukopenia, or hemorrhagic manifestations. The tourniquet test was not performed. Diagnosis of DHF required fever and all three of: hemorrhagic tendencies; thrombocytopenia (platelet <100 000/mm^3^); and evidence of plasma leakage (hematocrit change of ≥20% or clinical fluid accumulation or hypoproteinemia [serum protein 63 g/dL]). For DSS, DHF cases required either (i) tachycardia (pulse > 100/ minute) with narrow pulse pressure (<20 mmHg) or (ii) hypotension for age (systolic blood pressure <90 mmHg).

### WHO 2009 Classification

Based on WHO 2009 classification^9^, classification as probable dengue required fever with two or more of: nausea/vomiting, rash, aches/pains, leukopenia and any warning sign. Warning signs used were: abdominal pain/ tenderness, persistent vomiting (≥2 consecutive days), clinical fluid accumulation (pleural effusion or ascites on examination or radiography), mucosal bleeding, liver enlargement, and increase in hematocrit concurrent with rapid decrease in platelet count (interpreted as any hematocrit ≥20% over baseline with platelet <50000/mm^3^). Lethargy was not used as it was not routinely recorded in the dengue care path. For severe dengue, the criteria were: (i) plasma leakage (either clinical fluid accumulation or evidence of hematocrit change of ≥20%) and shock [with at least one of tachycardia (pulse > 100/minute), hypotension (systolic blood pressure <90 mmHg), or narrow pulse pressure (<20 mmHg)] or respiratory distress; severe bleeding was defined as WHO Grade 2 or above: hematemesis, melena, menorrhagia or clinical drop in hemoglobin requiring whole blood or packed red cell transfusion; severe organ involvement comprised hepatic injury (aspartate [AST] or alanine transaminase [ALT] levels > 1000 unit/L), renal impairment (Stage 2 Acute Kidney Injury^65^ defined as serum creatinine increase of 100% over baseline or calculated norm for age/gender/race), or encephalopathy. No dengue-related myocarditis was found in this cohort.

### Statistical methods

Univariate and multivariate conditional logistic regression were used to calculate crude and adjusted odds ratios (cOR, AcOR), respectively, and their 95% confidence intervals (CI) were used to assess the association of the variables with DHF. Confounding effect was minimized by performing multivariate conditional logistic regression adjusting for potential confounders identified in the univariate analysis. These potential confounders are exposures at initial presentation that were found to be statistically different (p < 0.05) between cases and controls in Table 1. Severe organ involvement is the disease outcome, which is a part of the disease severity (particularly for WHO 2009 classification), that we are interested to understand more in this study. One of the principles for confounding factors states that a confounding factor should not be in the (known) causal pathway of the outcome of interest. In addition, there is high collinearity between severe organ involvement (as outcome of interest) and severe dengue classification (as potential confounding factor) as shown in Table 3. As such, we did not perform statistical adjustment using disease severity as one of the confounding factors to achieve a more realistic interpretations. In Singapore, dengue infections were predominantly due to dengue serotype 1 (detected in 75% to 100% of dengue samples collected each month) during the epidemic in the year 2005 and 2006, and dengue serotype 2 (detected in up to 91% of dengue samples) during the epidemic in the year 2007 and 2008. Given that different dengue serotypes may cause different disease severity, each case was matched to 4–5 controls by epidemic year to minimize confounding effect due to different predominant dengue serotypes. All statistical analyses were performed using Stata 10.0 (Stata Corp., College Station, TX, 2005). All tests were conducted at the 5% level of significance, with OR, P-value and corresponding 95% CI reported where applicable.

### Ethics statement

The National Healthcare Group Domain Specific Review Board granted ethics approval of the study with a waiver of informed consent for collection of anonymized case note data and the data were analyzed anonymously (DSRB B/05/115, DSRB E/08/567).

## Additional Information

**How to cite this article**: Pang, J. *et al*. Diabetes, cardiac disorders and asthma as risk factors for severe organ involvement among adult dengue patients: A matched case-control study. *Sci. Rep.* **7**, 39872; doi: 10.1038/srep39872 (2017).

**Publisher's note:** Springer Nature remains neutral with regard to jurisdictional claims in published maps and institutional affiliations.

## Figures and Tables

**Table 1:** Demographic and comorbidities risk factors at first presentation of dengue patients with severe organ involvement outcome (Cases) compared to matched^ dengue patients with no severe organ involvement outcome (Controls).

	Controls^ (N = 865)	%	Cases (N = 174)	%	cOR	p-value	95% CI	95% CI	AcOR	p-value*	95% CI	95% CI
**Age (median)**	33	(24–42)	37.5	(27–52)	**1.03**	**<0.0001**	**1.02**	**1.04**	1.01	0.112	0.99	1.03
Age groups												
12–29	342	39.5	51	29.3	1				1			
30–39	241	27.9	45	25.9	1.25	0.318	0.81	1.93	1.25	0.318	0.8	1.95
40–49	167	19.3	30	17.2	1.17	0.531	0.72	1.9	1.07	0.792	0.62	1.72
50–59	77	8.9	18	10.3	1.58	0.128	0.88	2.84	1.11	0.751	0.52	1.99
≥60	38	4.4	30	17.2	**5.29**	**<0.0001**	**3**	**9.35**	**2.75**	**0.008**	**1.3**	**5.8**
Gender												
Female	347	40.1	84	48.3	**1.41**	**0.039**	**1.02**	**1.96**	1.7	0.09	0.95	1.93
Ethnic Groups												
Chinese	593	68.6	114	65.5	1				1			
Malay	84	9.7	20	11.5	1.26	0.403	0.74	2.14	1.09	0.76	0.62	1.93
Indians	86	9.9	17	9.8	1.03	0.915	0.59	1.8	1.15	0.647	0.64	2.07
Others	102	11.8	23	13.2	1.19	0.501	0.72	1.96	1.51	0.129	0.89	2.57
Epidemic Year^												
2005	585	67.6	117	67.2								
2006	115	13.3	23	12.2								
2007	75	8.7	16	9.2								
2008	90	10.4	18	10.3								
**DPF presentation**	5	(4–6)	5	(4–6)	**0.89**	**0.038**	**0.8**	**0.99**	0.93	0.186	0.83	1.04
**IgG positive**	241	93.8	70	98.6	1.85	0.579	0.21	16.23	0.88	0.917	0.08	10.2
Detection Assay												
Serology+	582	67.3	114	65.5	1				1			
PCR+	283	32.7	60	34.5	1.10	0.609	0.77	1.55	0.96	0.851	0.66	1.42
Any Pre-existing illness												
Yes	147	17	55	31.6	**2.22**	**<0.0001**	**1.53**	**3.22**	**1.63**	**0.023**	**1.07**	**2.49**
Number of Pre-existing illness												
0	718	83.0	117	67.2	1				1			
1	99	11.5	24	13.8	1.42	0.177	0.85	2.36	1.19	0.515	0.70	2.02
≥2	48	5.6	33	19.0	**3.92**	**<0.0001**	**2.45**	**6.29**	**2.90**	**<0.0001**	**1.66**	**5.07**
**Diabetes**	27	3.1	23	12.2	4.75	**<0.0001**	**2.63**	**8.56**	**2.21**	**0.027**	**1.1**	**5.02**
**Hypertension**	70	8.1	34	19.5	2.77	**<0.0001**	**1.77**	**4.33**	1.02	0.935	0.54	1.95
**Heart Failure**	2	0.2	0	0								
**Hyperlipidemia**	29	3.4	21	12.1	3.65	**<0.0001**	**2.03**	**6.57**	1.46	0.347	0.66	3.22
**Cardiac Disorder**	9	1	13	7.5	8.59	**<0.0001**	**3.41**	**21.66**	**4.30**	**0.009**	**1.45**	**12.78**
**Lung Disorder**	14	1.6	4	2.3	1.43	0.529	0.47	4.34	0.46	0.278	0.12	1.86
**Liver Disorder**	11	1.3	1	0.6	0.45	0.443	0.06	3.52	0.39	0.383	0.05	3.23
**Renal Disorder**	3	0.4	1	0.6	1.67	0.658	0.17	16.02	1.25	0.85	0.12	13.2
**Asthma**	34	3.9	13	7.5	**2.05**	**0.039**	**1.03**	**4.04**	**2.14**	**0.039**	**1.04**	**4.42**

DPF - Days post fever.

^^^Matched by year of presentation as the surrogate marker for predominant serotype to minimize the bias due to the effect of different predominant serotypes.

^*^Adjusted by age group, gender, day post fever duration at presentation, diabetic, hypertension, hyperlipidemia, cardiac disorder and asthma.

**Table 2:** The risk effect of severe organ involvement with two pre-existing comorbidities.

	Controls^ (N = 865)	%	Cases (N = 174)	%	cOR	p-value	95% CI	95% CI	AcOR	p-value*	95% CI	95% CI
**Diabetes, Cardiac disorder**												
No DM, no CD	831	96.07	144	82.76	1				1			
DM, no CD	25	2.89	17	9.77	**3.99**	**<0.0001**	**2.09**	**7.61**	**2.36**	**0.028**	**1.10**	**5.08**
CD, no DM	7	0.81	7	4.02	**6.73**	**0.001**	**2.18**	**20.83**	**4.85**	**0.011**	**1.43**	**16.41**
DM and CD	2	0.23	6	3.45	**18.93**	**<0.0001**	**3.74**	**95.74**	**8.02**	**0.019**	**1.40**	**45.92**
**Diabetes, Hypertension**												
No DM, no HT	783	90.52	133	76.44	1				1			
DM, no HT	12	1.39	7	4.02	**3.66**	**0.012**	**1.34**	**10.04**	2.37	0.131	0.77	7.26
HT, no DM	55	6.36	18	10.34	**1.96**	**0.024**	**1.09**	**3.51**	1.21	0.572	0.62	2.37
DM and HT	15	1.73	16	9.20	**5.95**	**<0.0001**	**2.85**	**12.43**	**2.68**	**0.035**	**1.07**	**6.68**
**Diabetes, Hyperlipidemia**												
No DM, no HL	779	90.06	136	78.16	1				1			
DM, no HL	10	1.16	3	1.72	1.86	0.362	0.49	7.08	1.50	0.562	0.38	5.94
HL, no DM	68	7.86	20	11.49	7.08	0.098	0.92	2.75	5.94	0.436	0.27	1.77
DM and HL	8	0.92	15	8.62	**10.89**	**<0.0001**	**4.41**	**26.85**	**4.25**	**0.014**	**1.34**	**13.52**
**Cardiac disorder, Hypertension**												
No CD, no HT	793	91.68	133	76.44	1				1			
CD, no HT	2	0.23	7	4.02	**21.83**	**<0.0001**	**4.44**	**107.30**	**15.79**	**0.002**	**2.87**	**86.76**
HT, no CD	63	7.28	28	16.09	**2.62**	**<0.0001**	**1.60**	**4.29**	1.41	0.275	0.76	2.61
CD and HT	7	0.81	6	3.45	**5.42**	**0.003**	**1.75**	**16.84**	2.64	0.141	0.73	9.57
**Cardiac disorder, Hyperlipidemia**												
No CD, no HL	829	95.84	146	83.91		1				1		
CD, no HL	7	0.81	7	4.02	**6.21**	**0.001**	**2.03**	**19.03**	**4.52**	**0.016**	**1.32**	**15.52**
HL, no CD	27	3.12	15	8.62	**2.80**	**0.002**	**1.44**	**5.44**	1.46	0.357	0.66	3.23
CD and HL	2	0.23	6	3.45	**17.28**	**0.001**	**3.44**	**86.91**	**5.79**	**0.046**	**1.03**	**32.64**

^^^Matched by year of presentation as the surrogate marker for predominant serotype to minimize the bias due to the effect of different predominant serotypes.

^*^Adjusted by age group, gender, day post fever duration at presentation, diabetic, hypertension, hyperlipidemia, cardiac disorder and asthma.

**Table 3:** Differential severity and clinical management among dengue patients with severe organ involvement outcome compared against matched dengue patients with no severe organ involvement outcome.

	Controls^ (N = 865)	%	Cases (N = 174)	%	AcOR	p-value	95% CI	95% CI
**Classification at Presentation**								
DHF/DSS (WHO 1997)	96	11.1	49	28.2	**3.11**	**<0.0001**	**2**	**4.85**
WS (WHO 2009)	567	65.6	125	71.8	1.38	0.95	0.95	2.02
**Classification as final outcome**								
DHF/DSS (WHO 1997)	167	19.3	81	46.6	**3.95**	**<0.0001**	**2.68**	**5.82**
WS (WHO 2009)	656	75.8	152	87.4	**1.93**	**0.008**	**1.19**	**3.14**
Severe dengue (WHO 2009)	104	12	174	100	**62.81**	**<0.0001**	**31.37**	**125.76**
**Liver involvement**	0	0	106	61				
**Renal involvement**	0	0	60	34.5				
**CNS involvement**	0	0	23	13.2				
**Renal & liver involvement**	0	0	15	8.6				
**Median LOS (IQR)**	4	(3–5)	5	(4–7)	**1.4**	**<0.0001**	**1.27**	**1.54**
**ICU admission**	0	0	12	6.9				
**IV Fluid**	748	86.5	167	96	**3.46**	**0.003**	**1.53**	**7.80**
**Blood transfusion**	4	0.5	10	5.8	**11.92**	**<0.0001**	**3.55**	**40.6**
**Platelets transfusion**	80	9.3	58	33.3	**5.12**	**<0.0001**	**3.21**	**8.16**

DHF - Dengue haemorrhagic fever.

DSS - Dengue shock syndrome.

WS - Dengue warning signs (WHO 2009 dengue classification).

CNS - Central nervous system.

LOS - Length of stay.

IQR - Interquartile range.

ICU - Intensive care unit.

IV - intravenous.

^Matched by year of presentation as the surrogate marker to minimize the impact of different predominant serotypes of that year.

*Adjusted by age group, gender, day post fever duration at presentation, diabetic, hypertension, hyperlipidemia, cardiac disorder and asthma.

**Table 4:** Differential clinical signs and symptoms at first presentation among dengue patients with severe organ involvement outcome compared against matched dengue patients with no severe organ involvement outcome.

	Controls^ (N = 865)	%	Cases (N = 174)	%	AcOR*	p-value	95% CI	95% CI
**Haemorrhagic manifestation**	394	45.6	77	44.3	0.96	0.833	0.68	1.36
**Rash**	413	47.8	75	43.1	0.92	0.66	0.65	1.31
**Leucopenia**	593	70.4	92	53.5	0.92	0.659	0.65	1.31
**Nausea/Vomiting**	604	69.8	135	77.6	**1.65**	**0.019**	**1.08**	**2.51**
**Aches pains**	655	75.7	135	77.6	1.23	0.327	0.81	1.87
**Abdominal pain**	226	26.1	70	40.2	**2.02**	**<0.0001**	**1.4**	**2.93**
**Cough**	188	51.2	36	48.7	0.94	0.852	0.50	1.76
**Persistent vomiting**	0	0	0	0				
**Clinical fluid accumulation**	1	0.1	3	1.7	**26.2**	**0.006**	**2.51**	**274.3**
**Mucosal bleeding**	199	23	37	21.3	1.02	0.936	0.67	1.56
**Lethargy**	289	33.4	55	31.6	0.85	0.39	0.58	1.23
**Hepatomegaly**	7	0.8	4	2.3	2.91	0.136	0.71	11.83
**Haemocrit ≥ 20% & Platelet < 50**	45	5.4	49	28.7	**6.67**	**<0.0001**	**3.98**	**11.17**
**Haemocrit ≥ 20%**	79	9.4	72	41.9	**5.91**	**<0.0001**	**3.89**	**8.98**
**Hypoproteinemia**	149	19.7	46	30.3	**1.86**	**0.007**	**1.18**	**2.94**
**Plasma leakage 2009**	80	9.3	73	42	**6.19**	**<0.0001**	**4.09**	**9.4**
**Thrombocytopenia**	731	86.9	163	95.3	**2.93**	**0.005**	**1.38**	**6.22**
**Tachycardia**	183	21.3	46	26.4	1.2	0.393	0.79	1.81
**Hypotension for age**	54	6.2	14	8.1	1.4	0.302	0.74	2.63
**Narrow pulse pressure**	3	0.4	2	1.2	2.2	0.422	0.32	15.08
**Shock**	221	25.6	55	31.6	1.24	0.271	0.84	1.83
**GIT bleed**	15	1.7	5	2.9	1.56	0.433	0.51	4.73
**Severe bleeding**	32	3.7	12	6.9	1.9	0.091	0.9	3.99
**Altered level of consciousness**	0	0	12	7.8				
**Jaundice**	6	0.7	1	0.6	1.1	0.934	0.12	9.78
**Median temperature (IQR)**	37.6	(36.9–38.4)	37.7	(37–38.4)	1.15	0.104	0.97	1.37
**Median blood pressure (IQR)**	103	(98–111)	103	(98–112)	**0.98**	**0.019**	**0.97**	**0.99**
**Median pulse (IQR)**	76	(67-87)	75	(65–84)	**0.99**	**0.035**	**0.97**	**0.99**

IQR - Interquartile range.

^^^Matched by year of presentation as the surrogate marker to minimize the impact of different predominant serotypes of that year.

^*^Adjusted by age group, gender, day post fever duration at presentation, diabetic, hypertension, hyperlipidemia, cardiac disorder and asthma.

**Table 5:** Differential clinical laboratory factors at first presentation among dengue patients with severe organ involvement outcome compared against matched dengue patients with no severe organ involvement outcome.

	Controls^ (N = 865)	Interquartile range	Cases (N = 174)	Interquartile range	AcOR*	p-value	95% CI	95% CI
**Median WBC**	2.7	(2–3.8)	3.3	(2.1–4.7)	**1.18**	**<0.0001**	**1.09**	**1.27**
**Median neutrophil**	53.8	(40.6–67.2)	61.7	(50–70)	**1.02**	**<0.0001**	**1.01**	**1.03**
**Median lymphocytes**	26	(18.5–35.2)	20.2	(15–28.9)	**0.96**	**<0.0001**	**0.95**	**0.98**
**Median monocytes**	10	(6.9–13.1)	8.6	(5.4–12.8)	0.97	0.14	0.94	1.01
**Median basophils**	0.2	(0–0.6)	0.2	(0–0.5)	0.89	0.541	0.61	1.3
**Median eosinophil**	0.2	(0–1)	0.1	(0–0.5)	**0.84**	**0.047**	**0.7**	**0.99**
**Median hematocrit**	43.1	(40–45.8)	44	(40–46.9)	**1.06**	**0.001**	**1.01**	**1.11**
**Median hemoglobin**	14.6	(13.5–15.6)	15	(13.6–16)	**1.2**	**0.006**	**1.06**	**1.37**
**Median platelet**	70	(47–87)	51	(25–73)	**0.98**	**<0.0001**	**0.98**	**0.99**
**Median sodium**	137	(134–139)	135	(132–137)	**0.84**	**<0.0001**	**0.8**	**0.88**
**Median potassium**	3.6	(3.3–3.9)	3.6	(3.3–4)	1.41	0.092	0.95	2.09
**Median urea**	3.4	(2.5–4.4)	4.3	(2.8–7.4)	**1.42**	**<0.0001**	**1.29**	**1.57**
**Median creatinine**	76	(62–88.5)	85	(65.3–128.3)	**1.03**	**<0.0001**	**1.02**	**1.04**
**Median bilirubin**	11	(8–15)	13	(9–18)	**1.06**	**<0.0001**	**1.04**	**1.09**
**Median AST**	112.5	(65–207.3)	771	(174–1242)	**1**	**<0.0001**	**1.004**	**1.006**
**Median ALT**	71	(39–148)	389	(91.5–717)	**1.006**	**<0.0001**	**1.005**	**1.008**
**Median protein**	68	(64–72)	66	(61–71)	**0.96**	**0.016**	**0.93**	**0.99**
**Median albumin**	39	(36–41)	37	(33–40)	**0.89**	**<0.0001**	**0.85**	**0.934**

WBC - White blood cells.

AST - Aspartate aminotransferases.

ALT - Alanine aminotransferases.

^^^Matched by year of presentation as the surrogate marker to minimize the impact of different predominant serotypes of that year.

^*^Adjusted by age group, gender, day post fever duration at presentation, diabetic, hypertension, hyperlipidemia, cardiac disorder and asthma.

## References

[b1] Shepard D. S., Undurraga E. A., Halasa Y. A. & Stanaway J. D. The global economic burden of dengue: a systematic analysis. Lancet Infect Dis. doi: 10.1016/S1473-3099(16)00146-8 (2016). 27091092

[b2] Stanaway J. D. . The global burden of dengue: an analysis from the Global Burden of Disease Study 2013. Lancet Infect Dis. doi: 10.1016/S1473-3099(16)00026-8 (2016). PMC5012511 26874619

[b3] Simmons C. P., Farrar J. J., Nguyen v V. & Wills B. Dengue. The New England journal of medicine 366, 1423–1432, doi: 10.1056/NEJMra1110265 (2012). 22494122

[b4] Bhatt S. . The global distribution and burden of dengue. Nature 496, 504–507, doi: 10.1038/nature12060 (2013). 23563266 PMC3651993

[b5] Gessner B. D. & Wilder-Smith A. Estimating the public health importance of the CYD-tetravalent dengue vaccine: Vaccine preventable disease incidence and numbers needed to vaccinate. Vaccine. doi: 10.1016/j.vaccine.2016.03.017 (2016). 27055020

[b6] Wills B. A. . Comparison of three fluid solutions for resuscitation in dengue shock syndrome. The New England journal of medicine 353, 877–889, doi: doi: 10.1056/NEJMoa044057 (2005). 16135832

[b7] Lam P. K. . Clinical characteristics of Dengue shock syndrome in Vietnamese children: a 10-year prospective study in a single hospital. Clinical infectious diseases: an official publication of the Infectious Diseases Society of America 57, 1577–1586, doi: 10.1093/cid/cit594 (2013). 24046311 PMC3814826

[b8] World Health Organisation. Dengue haemorrhagic fever: diagnosis, treatment, prevention and control. *2nd edition. Geneva. World Health Organisation* (1997).

[b9] World Health Organisation. Dengue: Guidelines for Diagnosis, Treatment, Prevention, and Control. *New edn. Geneva: TDR/World Health Organization* (2009). 23762963

[b10] Yacoub S. & Wills B. Predicting outcome from dengue. BMC medicine 12, 147, doi: 10.1186/s12916-014-0147-9 (2014). 25259615 PMC4154521

[b11] Leo Y. S. . Utility of warning signs in guiding admission and predicting severe disease in adult dengue. BMC infectious diseases 13, 498, doi: 10.1186/1471-2334-13-498 (2013). 24152678 PMC4015176

[b12] Thein T. L., Gan V. C., Lye D. C., Yung C. F. & Leo Y. S. Utilities and limitations of the World Health Organization 2009 warning signs for adult dengue severity. PLoS neglected tropical diseases 7, e2023, doi: 10.1371/journal.pntd.0002023 (2013). 23350013 PMC3547865

[b13] Alexander N. . Multicentre prospective study on dengue classification in four South-east Asian and three Latin American countries. Tropical medicine & international health: TM & IH 16, 936–948, doi: 10.1111/j.1365-3156.2011.02793.x (2011). 21624014

[b14] Seneviratne S. L., Malavige G. N. & de Silva H. J. Pathogenesis of liver involvement during dengue viral infections. Transactions of the Royal Society of Tropical Medicine and Hygiene 100, 608–614, doi: 10.1016/j.trstmh.2005.10.007 (2006). 16483623

[b15] Povoa T. F. . The pathology of severe dengue in multiple organs of human fatal cases: histopathology, ultrastructure and virus replication. PloS one 9, e83386, doi: 10.1371/journal.pone.0083386 (2014). 24736395 PMC3987999

[b16] Schmitz L. . Nonhematological organ dysfunction and positive fluid balance are important determinants of outcome in adults with severe dengue infection: a multicenter study from India. J Crit Care 26, 441–448, doi: 10.1016/j.jcrc.2011.05.008 (2011). 21737234

[b17] Woon Y. L. . A Two-Year Review on Epidemiology and Clinical Characteristics of Dengue Deaths in Malaysia, 2013–2014. PLoS neglected tropical diseases 10, e0004575, doi: 10.1371/journal.pntd.0004575 (2016). 27203726 PMC4874788

[b18] Yacoub S., Wertheim H., Simmons C. P., Screaton G. & Wills B. Cardiovascular manifestations of the emerging dengue pandemic. Nature reviews. Cardiology 11, 335–345, doi: 10.1038/nrcardio.2014.40 (2014). 24710495

[b19] Tan S. S. & Bujang M. A. The clinical features and outcomes of acute liver failure associated with dengue infection in adults: a case series. Braz J Infect Dis 17, 164–169, doi: 10.1016/j.bjid.2012.09.007 (2013). 23453417 PMC9427345

[b20] Ling L. M., Wilder-Smith A. & Leo Y. S. Fulminant hepatitis in dengue haemorrhagic fever. Journal of clinical virology: the official publication of the Pan American Society for Clinical Virology 38, 265–268, doi: 10.1016/j.jcv.2006.12.011 (2007). 17306619

[b21] Mallhi T. H. . Dengue-induced Acute Kidney Injury (DAKI): A Neglected and Fatal Complication of Dengue Viral Infection–A Systematic Review. Journal of the College of Physicians and Surgeons–Pakistan: JCPSP 25, 828–834, doi: 11.2015/JCPSP.828834 (2015). 26577971

[b22] Mallhi T. H. . Incidence, Characteristics and Risk Factors of Acute Kidney Injury among Dengue Patients: A Retrospective Analysis. PloS one 10, e0138465, doi: 10.1371/journal.pone.0138465 (2015). 26421839 PMC4589349

[b23] Carod-Artal F. J., Wichmann O., Farrar J. & Gascon J. Neurological complications of dengue virus infection. Lancet Neurol 12, 906–919, doi: 10.1016/S1474-4422(13)70150-9 (2013). 23948177

[b24] Solomon T. . Neurological manifestations of dengue infection. Lancet 355, 1053–1059, doi: 10.1016/S0140-6736(00)02036-5 (2000). 10744091

[b25] Rodrigues R. S. . Lung in dengue: computed tomography findings. PloS one 9, e96313, doi: 10.1371/journal.pone.0096313 (2014). 24836605 PMC4023925

[b26] Marchiori E. . Pulmonary hemorrhage syndrome associated with dengue fever, high-resolution computed tomography findings: a case report. Orphanet J Rare Dis 4, 8, doi: 10.1186/1750-1172-4-8 (2009). 19265524 PMC2660293

[b27] Laoprasopwattana K., Chaimongkol W., Pruekprasert P. & Geater A. Acute respiratory failure and active bleeding are the important fatality predictive factors for severe dengue viral infection. PloS one 9, e114499, doi: 10.1371/journal.pone.0114499 (2014). 25460594 PMC4252142

[b28] Huang S. Y., Lee I. K., Liu J. W., Kung C. T. & Wang L. Clinical features of and risk factors for rhabdomyolysis among adult patients with dengue virus infection. The American journal of tropical medicine and hygiene 92, 75–81, doi: 10.4269/ajtmh.14-0343 (2015). PMC4347396 25349377

[b29] Davis J. S. & Bourke P. Rhabdomyolysis associated with dengue virus infection. Clinical infectious diseases: an official publication of the Infectious Diseases Society of America 38, e109–111, doi: 10.1086/392510 (2004). 15156504

[b30] Halsey E. S. . Correlation of serotype-specific dengue virus infection with clinical manifestations. PLoS neglected tropical diseases 6, e1638, doi: 10.1371/journal.pntd.0001638 (2012). 22563516 PMC3341333

[b31] Global Burden of Disease Study, C. Global, regional, and national incidence, prevalence, and years lived with disability for 301 acute and chronic diseases and injuries in 188 countries, 1990–2013: a systematic analysis for the Global Burden of Disease Study 2013. Lancet 386, 743–800, doi: 10.1016/S0140-6736(15)60692-4 (2015). 26063472 PMC4561509

[b32] Toledo J. . Relevance of Non-communicable Comorbidities for the Development of the Severe Forms of Dengue: A Systematic Literature Review. PLoS neglected tropical diseases 10, e0004284, doi: 10.1371/journal.pntd.0004284 (2016). 26727113 PMC4699776

[b33] Thanachartwet V. . Identification of clinical factors associated with severe dengue among Thai adults: a prospective study. BMC infectious diseases 15, 420, doi: 10.1186/s12879-015-1150-2 (2015). 26468084 PMC4606996

[b34] Mallhi T. H. . Clinico-laboratory spectrum of dengue viral infection and risk factors associated with dengue hemorrhagic fever: a retrospective study. BMC infectious diseases 15, 399, doi: 10.1186/s12879-015-1141-3 (2015). 26423145 PMC4590689

[b35] Lee I. K., Liu J. W. & Yang K. D. Clinical and laboratory characteristics and risk factors for fatality in elderly patients with dengue hemorrhagic fever. The American journal of tropical medicine and hygiene 79, 149–153 (2008). 18689614

[b36] Rowe E. K. . Challenges in dengue fever in the elderly: atypical presentation and risk of severe dengue and hospital-acquired infection [corrected]. PLoS neglected tropical diseases 8, e2777, doi: 10.1371/journal.pntd.0002777 (2014). 24699282 PMC3974675

[b37] Trung D. T. . Clinical features of dengue in a large Vietnamese cohort: intrinsically lower platelet counts and greater risk for bleeding in adults than children. PLoS neglected tropical diseases 6, e1679, doi: 10.1371/journal.pntd.0001679 (2012). 22745839 PMC3383761

[b38] Pera A. . Immunosenescence: Implications for response to infection and vaccination in older people. Maturitas 82, 50–55, doi: 10.1016/j.maturitas.2015.05.004 (2015). 26044074

[b39] Chen W. H. . Vaccination in the elderly: an immunological perspective. Trends in immunology 30, 351–359, doi: 10.1016/j.it.2009.05.002 (2009). 19540808 PMC3739436

[b40] Martina B. E., Koraka P. & Osterhaus A. D. Dengue virus pathogenesis: an integrated view. Clinical microbiology reviews 22, 564–581, doi: 10.1128/CMR.00035-09 (2009). 19822889 PMC2772360

[b41] Htun N. S. . Is diabetes a risk factor for a severe clinical presentation of dengue?–review and meta-analysis. PLoS neglected tropical diseases 9, e0003741, doi: 10.1371/journal.pntd.0003741 (2015). 25909658 PMC4409149

[b42] Pang J. . Diabetes with hypertension as risk factors for adult dengue hemorrhagic fever in a predominantly dengue serotype 2 epidemic: a case control study. PLoS neglected tropical diseases 6, e1641, doi: 10.1371/journal.pntd.0001641 (2012). 22563519 PMC3341340

[b43] Pang J., Thein T. L., Leo Y. S. & Lye D. C. Early clinical and laboratory risk factors of intensive care unit requirement during 2004 inverted question mark2008 dengue epidemics in Singapore: a matched case inverted question markcontrol study. BMC infectious diseases 14, 649, doi: 10.1186/s12879-014-0649-2 (2014). 25475217 PMC4267742

[b44] Geerlings S. E. & Hoepelman A. I. Immune dysfunction in patients with diabetes mellitus (DM). FEMS Immunol Med Microbiol 26, 259–265 (1999). 10575137 10.1111/j.1574-695X.1999.tb01397.x

[b45] Hsueh W. A., Lyon C. J. & Quinones M. J. Insulin resistance and the endothelium. Am J Med 117, 109–117, doi: 10.1016/j.amjmed.2004.02.042 (2004). 15234647

[b46] Thein T. L. . Risk factors for fatality among confirmed adult dengue inpatients in Singapore: a matched case-control study. PloS one 8, e81060, doi: 10.1371/journal.pone.0081060 (2013). 24278377 PMC3838336

[b47] Martin R. K. Acute kidney injury: advances in definition, pathophysiology, and diagnosis. AACN Adv Crit Care 21, 350–356, doi: 10.1097/NCI.0b013e3181f9574b (2010). 21045572

[b48] Farooqi S. & Dickhout J. G. Major comorbid disease processes associated with increased incidence of acute kidney injury. World J Nephrol 5, 139–146, doi: 10.5527/wjn.v5.i2.139 (2016). 26981437 PMC4777784

[b49] Bravo J. R., Guzman M. G. & Kouri G. P. Why dengue haemorrhagic fever in Cuba? 1. Individual risk factors for dengue haemorrhagic fever/dengue shock syndrome (DHF/DSS). Transactions of the Royal Society of Tropical Medicine and Hygiene 81, 816–820 (1987). 3450004 10.1016/0035-9203(87)90041-1

[b50] Cunha R. V. . Dengue epidemic in the State of Rio Grande do Norte, Brazil, in 1997. Transactions of the Royal Society of Tropical Medicine and Hygiene 93, 247–249 (1999). 10492750 10.1016/s0035-9203(99)90008-1

[b51] Gonzalez D. . Classical dengue hemorrhagic fever resulting from two dengue infections spaced 20 years or more apart: Havana, Dengue 3 epidemic, 2001-2002. International journal of infectious diseases: IJID: official publication of the International Society for Infectious Diseases 9, 280–285, doi: 10.1016/j.ijid.2004.07.012 (2005). 16023878

[b52] Guzman M. G. . Dengue 2 virus enhancement in asthmatic and non asthmatic individual. Mem Inst Oswaldo Cruz 87, 559–564 (1992). 1343671 10.1590/s0074-02761992000400016

[b53] Holgate S. T. Innate and adaptive immune responses in asthma. Nat Med 18, 673–683, doi: 10.1038/nm.2731 (2012). 22561831

[b54] Wark P. A. . Asthmatic bronchial epithelial cells have a deficient innate immune response to infection with rhinovirus. The Journal of experimental medicine 201, 937–947, doi: 10.1084/jem.20041901 (2005). 15781584 PMC2213100

[b55] Contoli M. . Role of deficient type III interferon-lambda production in asthma exacerbations. Nat Med 12, 1023–1026, doi: 10.1038/nm1462 (2006). 16906156

[b56] Jartti T. & Korppi M. Rhinovirus-induced bronchiolitis and asthma development. Pediatr Allergy Immunol 22, 350–355, doi: 10.1111/j.1399-3038.2011.01170.x (2011). 21535176

[b57] Bosco A., Ehteshami S., Stern D. A. & Martinez F. D. Decreased activation of inflammatory networks during acute asthma exacerbations is associated with chronic airflow obstruction. Mucosal Immunol 3, 399–409, doi: 10.1038/mi.2010.13 (2010). 20336062 PMC2891355

[b58] O’Keefe J. H. & Bell D. S. Postprandial hyperglycemia/hyperlipidemia (postprandial dysmetabolism) is a cardiovascular risk factor. Am J Cardiol 100, 899–904, doi: 10.1016/j.amjcard.2007.03.107 (2007). 17719342

[b59] Cersosimo E. & DeFronzo R. A. Insulin resistance and endothelial dysfunction: the road map to cardiovascular diseases. Diabetes Metab Res Rev 22, 423–436, doi: 10.1002/dmrr.634 (2006). 16506274

[b60] Chaturvedi U. C. & Nagar R. Nitric oxide in dengue and dengue haemorrhagic fever: necessity or nuisance? FEMS Immunol Med Microbiol 56, 9–24, doi: 10.1111/j.1574-695X.2009.00544.x (2009). 19239490 PMC7110348

[b61] Thein T. L. . Association Between Increased Vascular Nitric Oxide Bioavailability and Progression to Dengue Hemorrhagic Fever in Adults. The Journal of infectious diseases 212, 711–714, doi: 10.1093/infdis/jiv122 (2015). 25732810

[b62] Pang J., Thein T. L., Leo Y. S. & Lye D. C. Early clinical and laboratory risk factors of intensive care unit requirement during 2004–2008 dengue epidemics in Singapore: a matched case-control study. BMC infectious diseases 14, 649, doi: 10.1186/s12879-014-0649-2 (2014). 25475217 PMC4267742

[b63] Low J. G. . The early clinical features of dengue in adults: challenges for early clinical diagnosis. PLoS neglected tropical diseases 5, e1191, doi: 10.1371/journal.pntd.0001191 (2011). 21655307 PMC3104968

[b64] Screaton G., Mongkolsapaya J., Yacoub S. & Roberts C. New insights into the immunopathology and control of dengue virus infection. Nat Rev Immunol 15, 745–759, doi: 10.1038/nri3916 (2015). 26603900

[b65] Mehta R. L. . Acute Kidney Injury Network: report of an initiative to improve outcomes in acute kidney injury. Crit Care 11, R31, doi: 10.1186/cc5713 (2007). 17331245 PMC2206446

